# Toward culturally responsive dementia recognition and assessment in Sub-Saharan Africa: lessons from empirical research in West Africa

**DOI:** 10.1093/geronb/gbag148

**Published:** 2026-07-22

**Authors:** Angélique Roquet, Kossi B Kounou, Paolo Martinelli, Daniela S Jopp

**Affiliations:** Institute of Psychology, University of Lausanne, Lausanne, Switzerland; Swiss Centre of Expertise in Life Course Research, LIVES Centre, Lausanne, Switzerland; Department of Applied Psychology, University of Lomé, Lomé, Togo; Psychopathological and Intercultural Clinics Laboratory (LCPI EA 4591), Jean Jaurès University, Toulouse, France; Institute of Psychology, University of Lausanne, Lausanne, Switzerland; Swiss Centre of Expertise in Life Course Research, LIVES Centre, Lausanne, Switzerland; Institute of Psychology, University of Lausanne, Lausanne, Switzerland; Swiss Centre of Expertise in Life Course Research, LIVES Centre, Lausanne, Switzerland

## Prevalence, trends, and barriers to early recognition of dementia in Low- and middle-income countries and Sub-Saharan Africa

Low- and middle-income countries (LMICs) are aging at an unprecedented pace. According to the World Health Organization, by 2050, 80% of older adults will live in these regions. Projections indicate that about 70% of all people living with dementia will reside in LMICs by 2050, largely due to demographic aging and population growth (Prince et al., 2015). In Sub-Saharan Africa (SSA), dementia prevalence currently varies from 2.3% (Nigeria) to 20.0% (Uganda), with incidence rates estimated at 13.3 per 1,000 person-years, equating to over 368,000 new cases annually. Despite this growing burden, most African countries lack adequate infrastructure for screening, diagnosis, treatment, and support (Akinyemi et al., 2022). Beyond these structural constraints, dementia is frequently interpreted through spiritual or supernatural lenses, which can lead to social exclusion or abuse (Spittel et al., 2019).

## Cultural and contextual barriers to early recognition: insights from *AfricAging*

The *AfricAging* project examined lay perceptions of aging in Switzerland, Morocco, and Togo. Among the 210 Togolese participants, aging was widely perceived as challenging, with 64.3% describing aging as a “burden,” 56.4% as “difficult to cope with,” and 52.6% as “not easy.” Qualitative narratives further illustrated that aging experiences are often framed through spiritual or religious explanations, such as ancestral punishment or demonic possession. These findings align with broader evidence from SSA, where supernatural causes (e.g., evil spirit) are used to explain dementia symptoms, particularly in rural and less-educated populations (Spittel et al., 2019, 2021).

Such beliefs are not limited to the general population and are also reported among some healthcare professionals and religious leaders (Spittel et al., 2019). In many communities, the absence of a specific term for dementia further contributes to misinterpretations of symptoms, hindering effective communication and early recognition (Mwendwa et al., 2022). Such attributions also carry significant social consequences, including stigma, exclusion, and abuse, that further delay care-seeking (Spittel et al., 2019). Such interpretations also shape care-seeking pathways for families and caregivers, who often turn first to traditional healers or faith-based providers rather than healthcare services, delaying diagnosis (Mwendwa et al., 2022).

Findings from *AfricAging* also suggest that mental health holds limited salience in public conceptions of aging in Togo. Only 2% of respondents in Togo, compared with 21% in Morocco and 29% in Switzerland, mentioned mental health as a component of aging well. Those who did so were young, urban adults engaged in academic or professional activities, suggesting a possible link with higher education levels and increased health literacy. This observation aligns with previous studies showing that higher health literacy, fostered by education and healthcare exposure, is associated with reduced belief in supernatural causes and less stigmatizing attitudes toward dementia (Spittel et al., 2021). Awareness campaigns and culturally sensitive education about age-related health changes and mental health issues are therefore strongly recommended to promote early help-seeking and to improve access to care.

Reliance on non-medical forms of care is not solely driven by stigma or misconceptions. It may also reflect deep-seated cultural values such as Ubuntu-type values of community and interdependence, emphasizing mutual care, solidarity, and collective responsibility (Metz, 2014). From this perspective, health and well-being are viewed as collective achievements sustained through family and communities (Jecker et al., 2022). *AfricAging* findings from Togo illustrate how these values translate into strong reliance on such networks as the first line of support when facing difficulties associated with aging or illness (Martinelli et al., under revision). This pattern reflects a deep trust in collective and spiritual forms of care rather than in healthcare systems, which are often perceived as distant, expensive, or culturally misaligned with local explanatory models of health (Banda, 2019; Martinelli et al., submitted). While this community-based reliance strengthens social cohesion, it may delay recognition of early cognitive or functional decline, leading to less reliable prevalence estimates for neurodegenerative diseases. These findings underscore the need to better integrate traditions and healthcare systems through culturally sensitive outreach and education strategies.

## Culturally responsive recommendations for research, policy, and practice

Taken together, these sociocultural interpretations and the lack of context-adapted cognitive screening tools hinder early dementia recognition in SSA. This results in a dual barrier: delayed recognition of symptoms within healthcare systems and a lack of culturally acceptable healthcare practices. Without adequate action, population aging will increase the number of undiagnosed cases, reinforcing stigma, social exclusion, and inequalities in access to care. Efforts to address these challenges must extend beyond medical interventions. Culturally sensitive public health messaging can help reduce stigma and promote early help-seeking. Targeted education of both communities and healthcare professionals can bridge explanatory gaps between biomedical and local models of illness. Stronger collaboration between formal healthcare services and community structures, including traditional healers and faith-based providers, is also essential for building trust and improving care pathways. Timely action is essential to support dementia detection, care, and prevention, and to support SSA’s healthcare systems in adapting to the ongoing and accelerating demographic transition. From a research perspective, these findings call for more empirical studies on dementia recognition in West Africa and culturally and structurally similar contexts, conducted with methodologically adapted approaches that account for the central role of cultural and social factors in illness representations and care-seeking behaviors, including community-based participatory methods and culturally and linguistically validated cognitive screening instruments.

## Data Availability

The present commentary is based on previously collected data from the *AfricAging* project. The datasets generated and/or analyzed during the study are not yet publicly available but will be made available upon reasonable request following acceptance of the associated publications. The study was not preregistered.
